# Steroid-resistant minimal change nephrotic syndrome associated with thymoma treated effectively with rituximab following thymectomy and cyclosporine: a case report

**DOI:** 10.1186/s12882-024-03485-2

**Published:** 2024-02-09

**Authors:** Yusaku Watanabe, Keiji Hirai, Momoko Hirata, Taisuke Kitano, Kiyonori Ito, Susumu Ookawara, Hisashi Oshiro, Yoshiyuki Morishita

**Affiliations:** 1 Division of Nephrology, First Department of Integrated Medicine, Saitama Medical Center, Jichi Medical University, 1-847 Amanuma-cho, Omiya-ku, Saitama-shi, Saitama-ken, 330-8503 Japan; 2 Department of Diagnostic Pathology, Saitama Medical Center, Jichi Medical University, Saitama, Japan

**Keywords:** Minimal change nephrotic syndrome, Rituximab, Thymoma, Myasthenia gravis

## Abstract

**Background:**

Minimal change nephrotic syndrome (MCNS) can be complicated by thymoma; however, no standard therapy for thymoma-associated MCNS has yet been established. We herein describe a case of steroid-resistant MCNS associated with thymoma, treated effectively with rituximab.

**Case presentation:**

A 71-year-old Japanese man was referred to our department with severe proteinuria (20 g/gCr). Renal biopsy showed minimal change disease and computed tomography revealed an anterior mediastinal mass. Based on these findings, he was diagnosed with thymoma-associated MCNS. He was treated with oral prednisolone (50 mg/day) and cyclosporine, and underwent thymectomy and plasma exchange. However, no improvement in proteinuria was observed. He therefore received intravenous rituximab 500 mg, resulting in a marked decrease in proteinuria from 5328 to 336 mg/day after 1 week.

**Conclusions:**

This case suggests that rituximab might be an effective therapy in patients with steroid-resistant MCNS associated with thymoma.

## Background

Thymoma is a rare mediastinal tumor that originates from the thymic epithelium and is known to be associated with autoimmune diseases, such as myasthenia gravis (MG) and pure red cell aplasia [1]. Thymoma may also rarely coexist with nephrotic syndrome, of which the most frequent type is minimal change nephrotic syndrome (MCNS) [2]. MCNS associated with thymoma is usually treated with corticosteroids, immunosuppressive drugs, and thymectomy; however, there is currently no established therapy [2]. Rituximab is a human monoclonal antibody that targets CD20 antigen expressed on B lymphocytes. Numerous studies reported that rituximab increased the remission rate and reduced the relapse rate in patients with steroid-resistant MCNS [3, 4], suggesting that it may also be effective for steroid-resistant MCNS complicated with thymoma [5]; however, reports of the efficacy of rituximab in patients with steroid-resistant MCNS associated with thymoma are lacking [5]. There is thus a need to accumulate such cases to determine the efficacy of rituximab in patients with thymoma-associated nephrotic syndrome. Here, we report the case of a patient with steroid-resistant MCNS associated with thymoma who was treated successfully with rituximab therapy.

## Case presentation

**Fig. 1:**
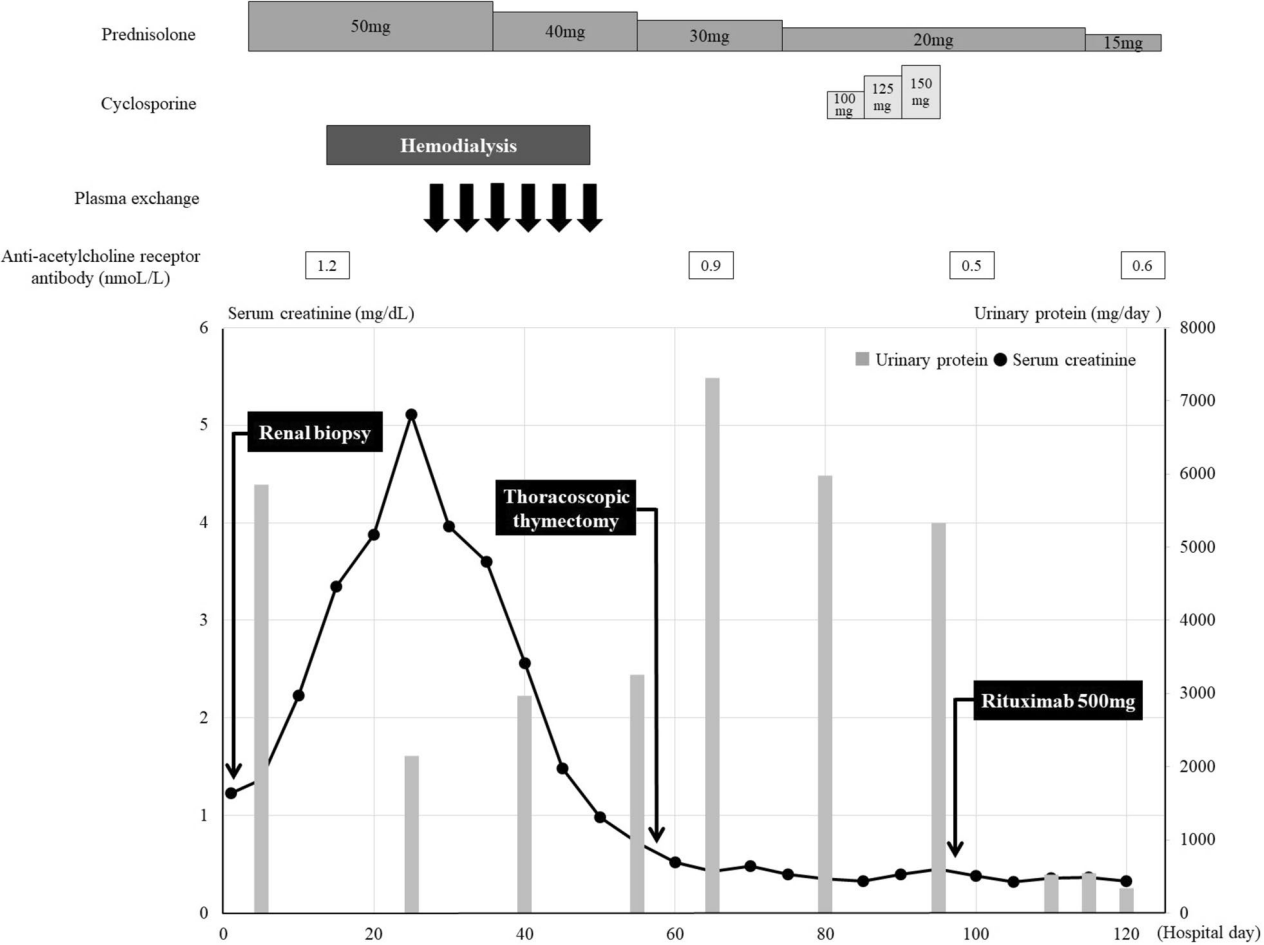
Patient’s clinical course. Horizontal axis shows number of days from admission; vertical axis shows serum creatinine and urinary protein. Anti-acetylcholine receptor antibody levels were decreased after thoracoscopic thymectomy; proteinuria improved after rituximab administration

**Fig. 2:**
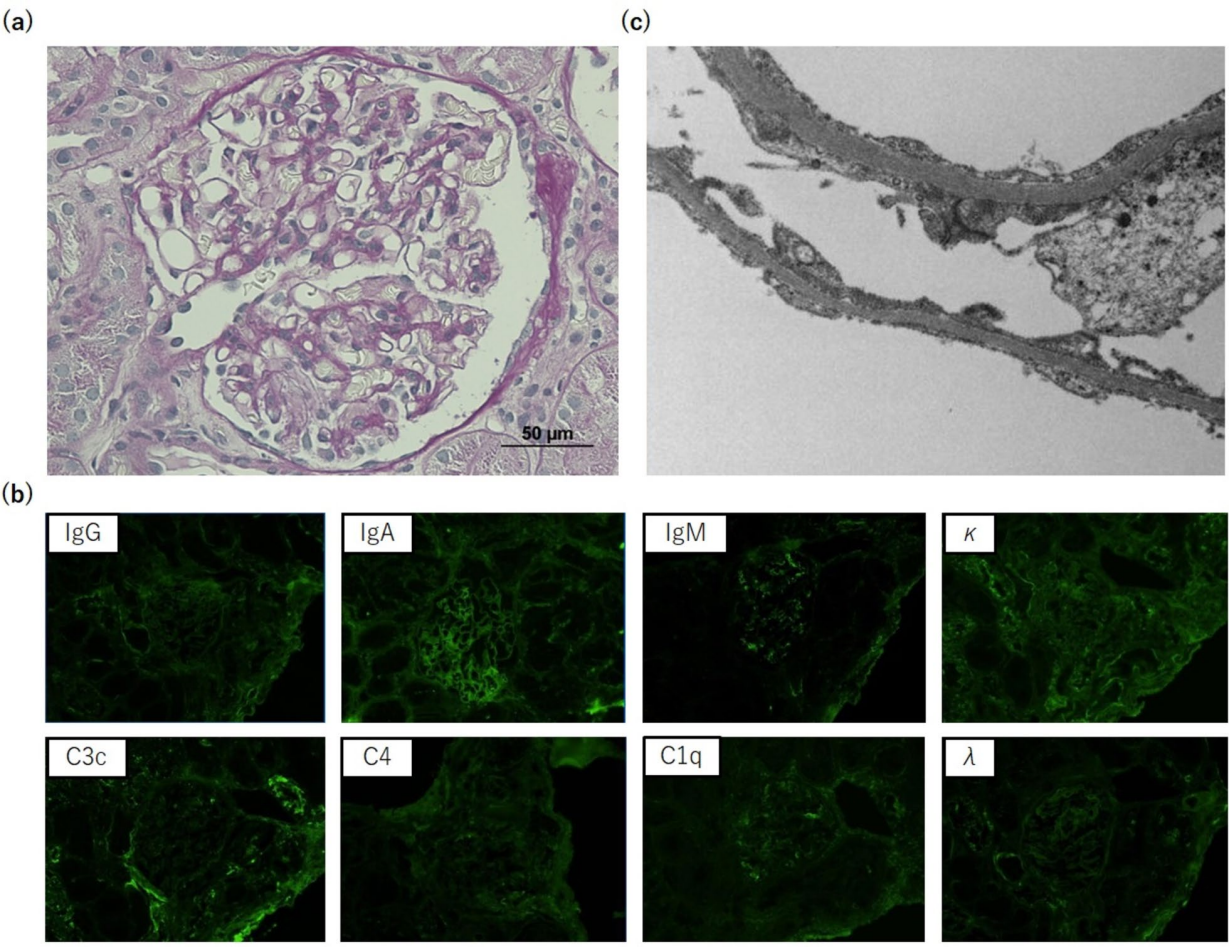
Renal biopsy findings. **a** Normal glomerulus without capillary wall or mesangial abnormalities (periodic acid-Schiff stain; magnification, ×400). **b** No significant staining for IgG, IgA, IgM, C3c, C4, C1q, kappa, and lambda (immunofluorescence stain; magnification, ×200). **c** Diffuse podocyte foot process effacement (uranyl acetate lead citrate stain; magnification, ×3000)

**Fig. 3:**
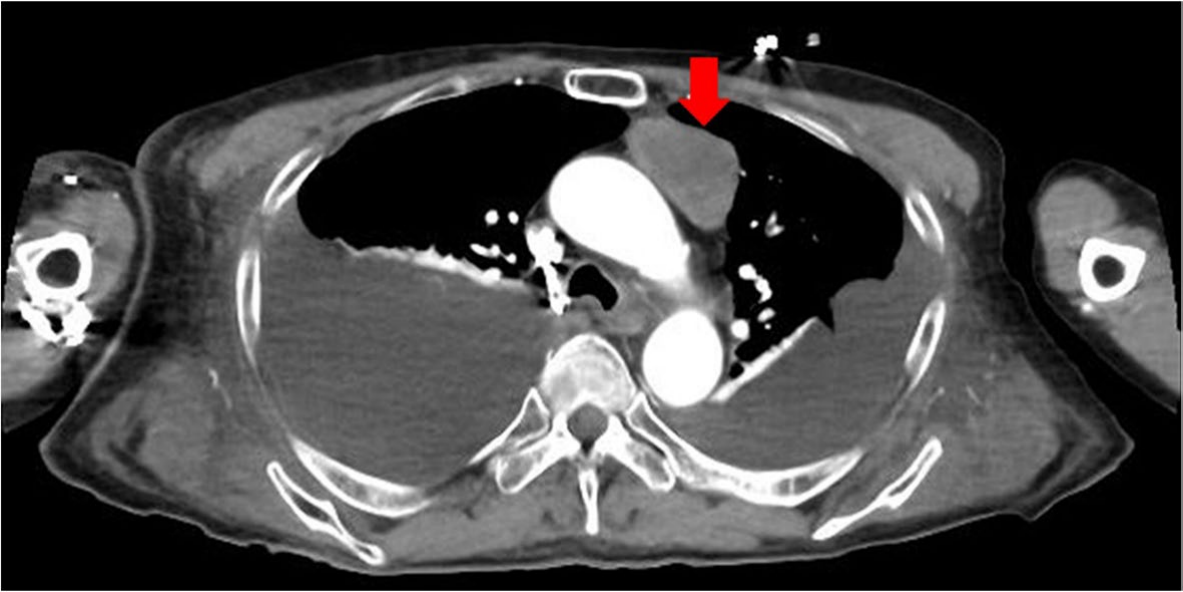
Computed tomography showed a low-attenuation anterior mediastinal mass not invading the surrounding organs

**Fig. 4:**
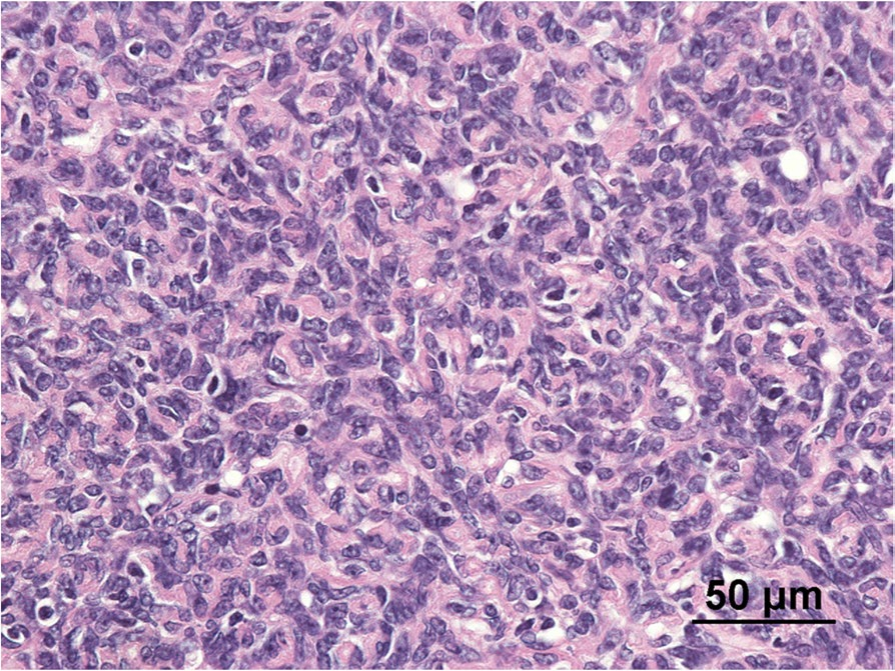
Microscopic findings of mediastinal tumor composed of spindle-shaped cells without nuclear pleomorphism (hematoxylin-eosin stain; magnification, ×400)

The patient was a 71-year-old Japanese man who had been treated for hypertension since the age of 67 years. He also had a history of left thalamic hemorrhage and was taking amlodipine 5 mg/day. Two weeks before admission, he developed right leg edema. He visited his general practitioner 7 days later and was found to have nephrotic syndrome, with serum albumin 1.0 g/L and proteinuria 20 g/gCr. He was subsequently referred to our department for further diagnostic workup. On admission, his blood pressure was 91/75 mmHg, heart rate was 76 beats per minute, respiratory rate was 21 breaths per minute, and oxygen saturation was 96% in room air. Coarse crackles were audible throughout both lung fields, and mild leg edema was detected. Laboratory data showed elevated serum creatinine (1.23 mg/dL), reduced estimated glomerular filtration rate (45.5 mL/min/1.73 m^2^), hypoalbuminemia (1.0 g/dL), hyperlipidemia (total cholesterol 484 mg/dL), and hypoglobulinemia (660 mg/dL). Urinalysis showed proteinuria of 9.20 g/gCr and a selectivity index of 0.17. Qualitative analysis of urinary Bence-Jones protein was positive, but immunofixation electrophoresis detected no monoclonal protein including Bence-Jones protein. Therefore, a bone marrow examination was not performed because false-positive results in qualitative analysis of urinary Bence-Jones protein are sometimes seen in cases of severe proteinuria [6]. The laboratory data at the time of referral are summarized in Table 1. Chest x-ray showed pleural effusion and an electrocardiogram showed no abnormalities. The patient was admitted to hospital for a renal biopsy. His clinical course after admission is depicted in Fig. 1. Oral prednisolone (50 mg/day) was initiated on hospital day 2, based on a clinical suspicion of MCNS, because of high-selectivity proteinuria. Light microscopy showed 28 glomeruli, with global sclerosis in three and mesangial proliferation in one. Immunofluorescence microscopy showed no significant staining for immunoglobulin, complement, or light chain. Electron microscopy showed diffuse podocyte foot process effacement. No deposition was observed in the glomerulus (Fig. 2). He was therefore diagnosed with MCNS. Dysarthria, dysphagia, and muscle weakness were observed on hospital day 12, and decreased consciousness and oxygenation levels developed on hospital day 14, when laboratory data showed acute kidney injury (serum creatinine 3.15 mg/dL, blood urea nitrogen 106 mg/dL). Hemodialysis was therefore started. Computed tomography revealed an anterior mediastinal mass (Fig. 3). The patient was also positive for anti-acetylcholine receptor antibody (1.2 nmoL/L). Based on his muscle weakness and positive anti-acetylcholine receptor antibodies (≥ 0.4 nmoL/L), the patient was diagnosed with MG. Plasma exchange therapy was initiated on hospital day 26 using 3480 mL of fresh frozen plasma as replacement and performed for a total of 6 treatments during 3 weeks, after which his urine output increased and his kidney function improved. Hemodialysis was discontinued on hospital day 48. After 4 weeks at the initial dose (50 mg/day), prednisolone was tapered to 40 mg/day for 2 weeks and then 30 mg/day for 2 weeks. The patient underwent a thoracoscopic thymectomy on hospital day 57, and histological analysis revealed spindle-shaped cells without nuclear pleomorphism, consistent with a diagnosis of type A thymoma (Fig. 4). His anti-acetylcholine receptor antibody had improved to 0.9 nmoL/L, but there was no improvement in his nephrotic-range proteinuria, dysarthria, dysphagia, or muscle weakness. After prednisolone was tapered to 20 mg/day, oral cyclosporine 100 mg/day was started on hospital day 81 and subsequently increased to 150 mg/day, but there was still no improvement in his proteinuria. He was therefore administered intravenous rituximab 500 mg on hospital day 96, after which, his proteinuria decreased markedly from 5328 to 336 mg/day and his anti-acetylcholine receptor antibody decreased to 0.5 nmoL/L. Nevertheless, there was still no improvement in his dysarthria, dysphagia, or muscle weakness. His prednisolone dose was tapered to 12.5 mg/day on hospital day 132, and he was transferred to another hospital for rehabilitation. After transfer, prednisolone was tapered to 10 mg/day for 4 weeks and then tapered by 1 mg every 4 weeks to a maintenance dose of 5 mg/day. Rituximab was not administered after the first dose. No relapse was observed during steroid tapering.

**Table 1:** Patient’s laboratory results at the time of referral to our department

Examination	Patient’s level	Reference range
Blood tests		
White blood cells (/µL)	9420	3900–9800
Neutrophils (%)	81.5	40–74
Lymphocytes (%)	13.9	19–48
Monocytes (%)	3.6	3.4–9.0
Eosinophils (%)	0.2	0–7
Basophils (%)	0.8	0–2
Red blood cells (×10^4^/µL)	582	377–555
Hemoglobin (g/dL)	18.7	12.0–17.6
Platelets (×10^4^/µL)	48.3	13.0–36.9
Total protein (g/dL)	4.2	6.4–8.2
Albumin (g/dL)	1.0	3.9–5.1
Total bilirubin (mg/dL)	0.44	0.2–1.0
Aspartate aminotransferase (IU/L)	27	11–30
Alanine aminotransferase (IU/L)	22	4–30
Total cholesterol (mg/dL)	484	142–248
LDL-cholesterol (mg/dL)	361	< 140
HDL-cholesterol (mg/dL)	73	48–103
Triglyceride (mg/dL)	250	30–117
Sodium (mEq/L)	131	138–145
Potassium (mEq/L)	3.8	3.6–4.8
Chloride (mEq/L)	97	100–110
Calcium (mg/dL)	7.4	8.6–10.1
Phosphate (mg/dL)	3.3	2.7–4.6
Blood urea nitrogen (mg/dL)	22	8–20
Creatinine (mg/dL)	1.23	0.65–1.07
eGFR (mL/min/1.73 m^2^)	45.5	≥ 60
C-reactive protein (mg/dL)	0.14	< 0.20
Blood glucose (mg/dL)	103	70–100
HbA1c (%)	5.5	4.6–6.2
IgG (mg/dL)	660	870–1700
IgA (mg/dL)	291	110–410
IgM (mg/dL)	69	33–190
IgE (IU/mL)	52	≤ 170
Antinuclear antibody	80	≤ 40
Urine tests		
pH	6.5	5.0–7.5
Specific gravity	1.035	1.005–1.030
Protein	3+	-
Glucose	3+	-
Red blood cells (/HPF)	5–9	1–4
White blood cells (/HPF)	1–4	1–4
Hyaline cast (/WF)	1–4	< 1
Epithelial cast (/WF)	1–4	< 1
Granular cast (/WF)	5–9	< 1
Fatty cast (/WF)	5–9	< 1
BJP	+	-
Monoclonal protein	-	-
Urinary protein (g/g Cr)	9.20	< 0.15
Selectivity index	0.17	
N-acetyl-β-D-glucosaminidase (IU/L)	176.1	≤ 7
β2 microglobulin (µg/L)	4997	≤ 230

eGFR was calculated using a modified version of the Modification of Diet in Renal Disease formula of the Japanese Society of Nephrology: eGFR (mL/min/1.73 m^2^) = 194 × ^−0.287^ × serum creatinine^−1.094^ (× 0.739 for women)

*Abbreviations: BJP *Bence-Jones protein, *eGFR *Estimated glomerular filtration rate, *HbA1c *Hemoglobin A1c, HDL High-density lipoprotein, *HPF *High-power field, *WF *Whole field, *IgA *Immunoglobulin A, *IgG *Immunoglobulin G, *IgM *Immunoglobulin M, *IgE *Immunoglobulin E, *LDL *Low-density lipoprotein

## Discussion and conclusions

Thymoma is known to be associated with various autoimmune diseases, including MG, red cell aplasia, and systemic lupus erythematosus, with MG occurring in approximately 44% of patients with thymoma [1]. Thymoma can also rarely be concurrent with nephrotic syndrome (about 1% of cases), usually minimal change disease [2]. The current patient was considered to have thymoma complicated with MG and MCNS, based on the coincident occurrence of an anterior mediastinal mass, massive proteinuria, and muscle weakness.

MCNS is usually treated with high-dose corticosteroids as first-line therapy [7], with additional thymectomy in cases complicated by thymoma [2]. However, there is currently no standard therapy for minimal change disease associated with thymoma [2]. The present patient’s proteinuria did not improve after thymectomy followed by corticosteroid for 12 weeks and cyclosporine for 2 weeks; however, urinary protein excretion was markedly reduced 11 days after rituximab administration. Previous report showed partial remission 1 week after and complete remission 3 weeks after administration of rituximab, largely consistent with our case [5]. Previous report showed that rituximab was effective in thymoma-associated MCNS which was resistant to corticosteroid and thymectomy [5]. Our case showed that rituximab was effective in thymoma-associated MCNS which was resistant to cyclosporine in addition to corticosteroid and thymectomy. The advantage of our case is that we have shown that rituximab is effective in thymoma-associated MCNS refractory to multimodal therapy with corticosteroid, thymectomy, and cyclosporine. Rituximab played a significant role in this case; however, the possibility cannot be excluded that cyclosporine and thymectomy may have affected the effect of rituximab. Further case accumulation is necessary to clarify the efficacy of rituximab in patients with steroid-resistant MCNS associated with thymoma. Rituximab is usually administered every 6 months for steroid-resistant MCNS [8]. In our case, however, rituximab was not administered after the first dose, because he was at high risk of infection due to low body mass index (14.8 kg/m^2^) [9].

Autoantibodies against nephrin, an essential component of the slit diaphragm, have recently been reported as the primary cause of minimal change disease [10], while anti-annexin A2 and anti-ubiquitin carboxy-terminal hydrolase L1 antibodies have also been reported as possible factors contributing to the development of minimal change disease [11, 12]. In the setting of thymoma, thymoma cells stimulate antibody-producing B-cell clones to produce various autoantibodies [13]. In the current case, rituximab was thus thought to have reduced proteinuria by suppressing these autoantibodies through B-cell depletion [14].

Rituximab has been shown to improve clinical symptoms and decrease anti-acetylcholine receptor antibodies in patients with MG and thymoma [15]. Notably however, dysphagia and muscle weakness failed to improve after rituximab administration in the present case. There are several possible reasons why rituximab failed to improve MG in this case: first, the dose of rituximab may have been inadequate, given that anti-acetylcholine receptor antibodies did not decrease after rituximab initiation, and second, the progression of sarcopenia caused by steroid-induced myopathy, immobility during prolonged hospitalization, nephrotic syndrome, and poor dietary intake might have weakened the therapeutic effect of rituximab.

In conclusion, we report a patient with steroid-resistant MCNS associated with thymoma who was treated successfully with rituximab. Rituximab might thus be an effective therapy for patients with steroid-resistant MCNS associated with thymoma.

## Data Availability

No datasets were generated or analysed during the current study.

## Abbreviations

MCNS: Minimal change nephrotic syndrome
MG: myasthenia gravis
